# Barriers and enablers to implementing antenatal magnesium sulphate for fetal neuroprotection guidelines: a study using the theoretical domains framework

**DOI:** 10.1186/s12884-015-0618-9

**Published:** 2015-08-18

**Authors:** Emily Bain, Tanya Bubner, Pat Ashwood, Emer Van Ryswyk, Lucy Simmonds, Sally Reid, Philippa Middleton, Caroline A. Crowther

**Affiliations:** Australian Research Centre for Health of Women and Babies, Robinson Research Institute, Discipline of Obstetrics and Gynaecology, School of Medicine, The University of Adelaide, Adelaide, South Australia Australia; The Women’s and Children’s Hospital, North Adelaide, South Australia Australia; Liggins Institute, The University of Auckland, Auckland, New Zealand

## Abstract

**Background:**

Strong evidence supports administration of magnesium sulphate prior to birth at less than 30 weeks’ gestation to prevent very preterm babies dying or developing cerebral palsy. This study was undertaken as part of The WISH (Working to Improve Survival and Health for babies born very preterm) Project, to assess health professionals’ self-reported use of antenatal magnesium sulphate, and barriers and enablers to implementation of 2010 Australian and New Zealand clinical practice guidelines.

**Methods:**

Semi-structured, one-to-one interviews were conducted with obstetric and neonatal consultants and trainees, and midwives in 2011 (*n* = 24) and 2012–2013 (*n* = 21) at the Women’s and Children’s Hospital, South Australia. Transcribed interview data were coded using the Theoretical Domains Framework (describing 14 domains related to behaviour change) for analysis of barriers and enablers.

**Results:**

In 2012–13, health professionals more often reported ‘routinely’ or ‘sometimes’ administering or advising their colleagues to administer magnesium sulphate for fetal neuroprotection (86 % in 2012–13 vs. 46 % in 2011). ‘Knowledge and skills’, ‘memory, attention and decision processes’, ‘environmental context and resources’, ‘beliefs about consequences’ and ‘social influences’ were key domains identified in the barrier and enabler analysis. Perceived barriers were the complex administration processes, time pressures, and the unpredictability of preterm birth. Enablers included education for staff and women at risk of very preterm birth, reminders and ‘prompts’, simplified processes for administration, and influential colleagues.

**Conclusions:**

This study has provided valuable data on barriers and enablers to implementing magnesium sulphate for fetal neuroprotection, with implications for designing and modifying future behaviour change strategies, to ensure optimal uptake of this neuroprotective therapy for very preterm infants.

**Electronic supplementary material:**

The online version of this article (doi:10.1186/s12884-015-0618-9) contains supplementary material, which is available to authorized users.

## Background

Preterm birth (birth at less than 37 completed weeks’ gestation), accounts for three quarters of perinatal mortality and over half of long-term morbidity [1]. While survival rates for preterm infants have increased substantially over time, infants remain at risk of a wide range of complications in the neonatal period and in the long-term, including neurodevelopmental impairments such as cerebral palsy [1, 2]. Cerebral palsy is the most common physical disability in childhood, with an estimated prevalence of around two per 1000 live births in Australia; despite research and clinical advances, its incidence remains stable [3]. There is a clear and established association between cerebral palsy and preterm birth, with over 40 % of individuals with cerebral palsy born preterm [3]. Accordingly, prevention of cerebral palsy for infants born preterm is of high interest, with questions relating to prevention identified as research priorities by consumers, clinicians and researchers [4].

Over two decades ago, a case control study described an association between maternal receipt of magnesium sulphate and a reduction in cerebral palsy for very low birthweight infants [5]. Subsequent to this and other observational studies, five randomised controlled trials were conducted assessing antenatally administered magnesium sulphate for preventing cerebral palsy [6–10]. These five trials were included in a Cochrane systematic review, which confirmed benefit, showing that 63 mothers need to be given magnesium sulphate prior to very preterm birth to prevent one case of cerebral palsy [11]. In response to these findings, in many countries, professional bodies have recommended this therapy. In 2010 an Australian and New Zealand Guideline Development Panel prepared and published National Health and Medical Research Council (NHMRC) endorsed clinical practice guidelines (see Additional file 1) [12]. Even with these guidelines, however, it was not anticipated that health professionals would immediately begin using this therapy without some form of active implementation [13, 14]. Based on knowledge of interventions likely to increase uptake of evidence into obstetric practice, an implementation project WISH (Working to Improve Survival and Health for babies born very preterm) was planned, and funded by the Cerebral Palsy Alliance Research Foundation [15]. This Project is ongoing and comprises a package of active implementation strategies to guide the introduction and local adaptation of guideline recommendations, and to monitor and improve uptake and health outcomes [15].

While interventions to assist implementing evidence into obstetric practice have been identified [16], recognising and targeting local, context specific barriers and enablers is important for successful knowledge translation. Implementation researchers have highlighted benefits of identifying the theoretical domains that are causally related to behaviours to assist with appropriately targeting interventions [17, 18]. Through consensus, behavioural experts have identified a set of 14 domains within a Theoretical Domains Framework (TDF) that may be considered when assessing implementation problems and used as a basis for intervention development (see Table 1) [17, 18]. These domains have been used to understand implementation difficulties across a range of healthcare settings and conditions, such as in the assessment of barriers and enablers to implementing preconception care guidelines by general practitioners [19], pregnancy weight management and obesity guidelines by health professionals [20], and for engaging with pregnant women about stopping smoking [21]. These theoretical domains can potentially be used to identify aspects of health professionals’ behaviour, which can be targeted to promote use of antenatal magnesium sulphate for fetal neuroprotection in line with guideline recommendations.

**Table 1:** Domains of the theoretical domains framework, adapted from Cane et al. [17]

Domain	Definition
1 Knowledge	An awareness of the existence of something
2 Skills	An ability or proficiency acquired through practice
3 Social/professional role and identity	A coherent set of behaviours and displayed personal quality of an individual in a social or work setting
4 Beliefs about capabilities	Acceptance of the truth, reality, or validity about an ability, talent or facility that a person can put to constructive use
5 Optimism	The confidence that things will happen for the best or that desired goals will be attained
6 Beliefs about consequences	Acceptance of the truth, reality, or validity about outcomes of a behaviour in a given situation
7 Reinforcement	Increasing the probability of a response by arranging a dependent relationship, or contingency, between the response and a given stimulus
8 Intentions	A conscious decision to perform a behaviour or a resolve to act in a certain way
9 Goals	Mental representations of outcomes or end states that an individual wants to achieve
10 Memory, attention and decision processes	The ability to retain information, focus selectively on aspects of the environment and choose between two or more alternatives
11 Environmental context and resources	Any circumstance of a person’s situation or environment that discourages or encourages the development of skills and abilities, independence, social competence, and adaptive behaviour
12 Social influences	Those interpersonal processes that can cause individuals to change their thoughts, feelings, or behaviours
13 Emotion	A complex reaction pattern, involving experiential, behavioural, and physiological elements, by which the individual attempts to deal with a personally significant matter or event
14 Behavioural regulation	Anything aimed at managing or changing objectively observed or measured actions

In parallel to this study, as part of The WISH Project, we have been examining the local uptake of antenatal magnesium sulphate before and following publication of the NHMRC endorsed guidelines at a tertiary maternity hospital, the Women’s and Children’s Hospital (WCH) in South Australia [22]. The aims of this study were to assess health professionals’ self-reported use and knowledge of bi-national guidelines for antenatal magnesium sulphate for fetal neuroprotection over time, and to explore local barriers and enablers to implementation using theoretical domains related to behaviour change.

## Methods

### Design

Semi-structured interviews, with analysis of barriers and enablers guided by the TDF [17].

### Participants and setting

Participants were obstetric and neonatal consultants and trainee medical officers, as well as midwives at the WCH. The first phase of interviews was conducted from May to August 2011 (following publication of the NHMRC endorsed guidelines in March 2010 and prior to dissemination of The WISH Project materials), and the second phase of interviews was undertaken between August 2012 and February 2013. The WCH is the largest tertiary maternity centre in Adelaide, South Australia, with almost 5000 births per annum and a 14 bed Neonatal Intensive Care Unit, which is responsible for providing intensive care to babies born at the WCH, elsewhere in South Australia, and at times, the Northern Territory, Western Victoria and far west of New South Wales, Australia [23].

### Procedure

For both time periods, contact details of consultant and trainee obstetricians, neonatologists and midwives were obtained and numbered consecutively. In 2011, it was pre-specified that ten obstetricians, eight midwives, and six neonatologists would be interviewed (from totals of 62, 82 and 20 respectively). Sets of random numbers were computer-generated and the corresponding individuals invited to participate via email. In 2012–13 it was similarly pre-specified that ten obstetricians, eight midwives and six neonatologists would be interviewed (from total numbers of 64, 53 and 20 respectively) (the total number of midwives decreased in 2012–13, as contact details of those rostered to the postnatal ward were not provided). If an individual declined, an additional random number was computer-generated and the corresponding staff member was invited to participate. In the case where an individual who participated in 2011 was again randomly selected in 2012–13, a further random number was computer-generated. Staff wishing to participate opted into the study by signing a consent form for recording of interviews and use of anonymised quotations.

Participants took part in single interviews with one of five researchers (EB, EVR, LS, SR, TB). Four of the interviewers (EB, EVR, LS, TB) were health researchers who were not employed by the WCH and did not work on a day-to-day basis with the participants. External to her research role, SR was employed as an obstetric trainee medical officer. Where possible, interviews were conducted face-to-face in a research office within the hospital or within participants’ work offices (two interviews were conducted by telephone). An interview schedule with questions guided the discussion. The interviews were audio-taped and transcribed verbatim. Transcriptions were cross-checked against the recordings for accuracy and de-identification.

### Interview schedule

The interview schedule (see Additional file 2) was developed primarily to elicit responses about health professionals’ self-reported use and knowledge of the 2010 bi-national clinical practice guidelines, and barriers and enablers to implementation. Participants were first asked to describe their position and experience (length of service). Participants were then asked a series of closed and open-ended questions to explore their and their colleagues’ practices regarding administration or advising administration of antenatal magnesium sulphate, their knowledge of guideline recommendations, benefits of therapy, and concerns regarding adverse effects. Participants were asked to consider barriers and enablers to implementation, and views regarding possible solutions or ways forward. The interview schedule was piloted with three health professionals (one obstetrician, one neonatologist, and one midwife) prior to data collection to assess its practicability and acceptability.

### Analysis

Participants’ responses to questions relating to their knowledge and use of antenatal magnesium sulphate, its benefits and adverse effects, were coded and have been summarised narratively and using percentages. The responses were summarised across all professional groups, given that no apparent differences between groups were observed. Content analysis related to barriers and enablers was conducted following a framework approach [24], and guided by the TDF [17]. The generated themes correlated strongly with the domains (see Table 1) [17], and as such, relevant participant responses/quotes were coded by domain, and copied and pasted into an Excel spreadsheet of domains, stratified by professional group and whether the statement related to a perceived barrier, enabler or both. The spreadsheets were reviewed and statements compared between and across domains, professional groups, and the two study periods. Two investigators (EB and TB) read and re-read all of the transcripts, and independently coded these. The analysis was discussed between researchers to reach agreement.

### Ethics

This study was approved by the WCH Children, Youth and Women’s Health Service Human Research Ethics Committee (REC2304/8/13).

## Results

In 2011, 24 health professionals participated: ten obstetricians, eight midwives and six neonatologists; in 2012–13, there were 21 participants: eight obstetricians, eight midwives and five neonatologists. In 2011, one midwife declined participation, and in 2012–13, one obstetric consultant and one neonatal trainee declined. In 2012–13, there were three fewer participants than original planned (with one less neonatal trainee, obstetric trainee and obstetric consultant) due to difficulties contacting those participants and scheduling interview times. Final participant characteristics are summarised in Table 2. Interviews were of mean 12 ± 5 min duration (range 6 to 26 min).

**Table 2:** Participant characteristics

Participant group		Total	Length of service at WCH (years)			
			<1	1–5	5–10	>10
2011		24				
Obstetrician	Consultant	5			2	3
	Trainee medical officer	5	4	1		
Neonatologist	Consultant	3		1	1	1
	Trainee medical officer	3	1	1	1	
Midwife		8		1	1	6
2012–13		21				
Obstetrician	Consultant	4			2	2
	Trainee medical officer	4		4		
Neonatologist	Consultant	3			1	2
	Trainee medical officer	2		1	1	
Midwife		8		1	1	6

*WCH* Women’s and children’s hospital

### Knowledge and use of antenatal magnesium sulphate

**Fig. 1:**
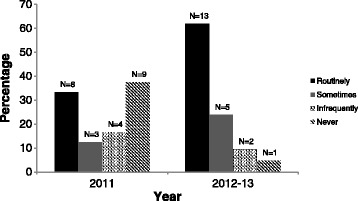
Summary of answers by year to the question: ‘Do you administer (or advise your colleagues to administer) magnesium sulphate to women at risk of preterm birth?

**Fig. 2:**
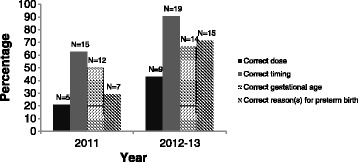
Summary by year indicating percentage of participants that correctly specified antenatal magnesium sulphate guideline recommendations (regarding: dose, timing, gestational age, and reasons for preterm birth)

Participants in 2012–13, compared with those in 2011, were more likely to report that they ‘*Routinely’* or ‘*Sometimes’* administered or advised their colleagues to administer antenatal magnesium sulphate for fetal neuroprotection (Fig. 1). All participants (21/21) in 2012–13 and 92 % (22/24) in 2011 believed that their colleagues administered/advised administration of this therapy. The majority of participants in 2012–13 (17/21, 85 %) and 2011 (18/24, 75 %) reported being aware of the bi-national clinical practice guidelines. In 2012–13, compared with in 2011, participants were more often able to correctly cite specific bi-national guideline recommendations regarding dosage, timing prior to birth, gestational age, and reason(s) for preterm birth (see Fig. 2).

### Benefits and adverse effects of antenatal magnesium sulphate

**Fig. 3:**
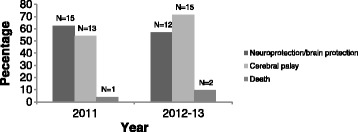
Summary by year indicating percentage of participants who specified neuroprotection/brain protection, reductions in cerebral palsy and death as benefits of antenatal magnesium sulphate

In both 2012–13 and 2011 over half of participants specifically discussed ‘brain protection’ or ‘neuroprotection’, and/or a reduction in cerebral palsy as benefits of antenatal magnesium sulphate therapy (see Fig. 3). Across both time periods, very few participants cited a combined reduction in neonatal death and cerebral palsy as a benefit of treatment.

**Fig. 4:**
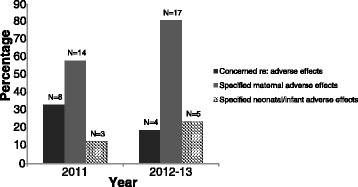
Summary by year indicating percentage of participants who were concerned about and/or who specifically mentioned maternal and/or neonatal/infant adverse effects of antenatal magnesium sulphate

In 2012–13 approximately one fifth of participants expressed that they had concern regarding adverse effects of therapy, compared with approximately one third in 2011. Though often not ‘of concern’, a number of participants cited possible adverse effects for the mother, however far fewer (and predominately neonatologists) discussed potential neonatal or infant adverse effects (see Fig. 4).

### Barriers and enablers

Barriers and enablers to the use of antenatal magnesium sulphate for fetal neuroprotection across both time periods, as perceived by obstetricians, neonatologists and midwives, were primarily related to theoretical domains: ‘knowledge’, ‘skills’, ‘memory, attention and decision processes’, ‘environmental context and resources’, ‘beliefs about consequences’, and ‘social influences’. These domains are discussed first, followed by domains that were less prominent: ‘professional role and identity’ and ‘reinforcement’. Statements depicting the theme for each domain have been chosen accordingly. Only those theoretical domains found to be relevant are presented.

### Knowledge and skills

The closely aligned theoretical domains ‘knowledge’ and ‘skills’ refer to whether individuals are aware of an intervention, and their ability (acquired through practice) to perform the intervention. Across both time periods (2011 and 2012–13), each professional group cited information dissemination to increase knowledge of and skills for the use of magnesium sulphate for fetal neuroprotection (such as through in-service training and pamphlets/brochures for health professionals and women) as a key enabler. In 2011, several staff members highlighted lack of awareness as a barrier: *“I guess just that everybody, you know, has this information, which I obviously didn’t.” (MW6-2011).*

In 2011, each professional group identified a need for staff education, and also education for pregnant women at risk of giving birth very preterm:

*“it’s really education… [for] those who might be in a position to manage a woman at risk of having an extremely preterm baby… for physicians and midwives… and also education for pregnant women” (CN2-2011).*

In regards to skills, in 2011, unfamiliarity with the medication was identified as a potential barrier. Correspondingly, training was identified, particularly by midwives and also by neonatologists, as an enabler.

In 2012–13, there was recognition, particularly by midwives, of increased knowledge and skills for appropriate administration:

*“I think we were a bit gung ho initially, whereas now we’ve got a better understanding of it… I just think we’re better at recognising and administering it” (MW2-2012/13).*

Each professional group discussed continued education, to reinforce knowledge, as enabling, and emphasised the need for education for new groups of staff:

*“… it just has to be ongoing education and more awareness… it’s a continuing process, isn’t it? With more new staff, at all levels starting from midwives, to new registrars, and among consultants” (TN2-2012/13).*

During 2012–13, a number of midwives similarly highlighted the importance of educating women at risk of very preterm birth.

### Memory, attention and decision processes

This theoretical domain relates to the factors that influence whether an individual remembers and chooses to engage in an intervention. Across both time periods staff from each professional group, and particularly obstetricians, highlighted not *“thinking of it”*, or not *“remembering it”* as a barrier. *“Forgetting”* was often perceived to be a consequence of being in a ‘rush’, or infrequent use:

*“You know what the biggest problem is… to remember to do it… sometimes we’re in so much of a hurry, right, to do something, that we forget to do it” (CO2-2012/13).*

Across both time periods, prompts or systems to remind staff to prescribe, administer or advise antenatal magnesium sulphate were seen as important enablers for increasing use. In 2011, several neonatologists and particularly obstetricians suggested benefits of ‘check-list approaches’, and also visual reminders and signage (such as posters in drug rooms and the labour ward, and stamps or ‘alerts’ in the front of medical records):

*“… a few more visual reminders… you know, kind of ‘Have you thought about?’ … If it’s in our face, we’ll think about it. If it’s not, we’ll forget” (TO2-2011).*

Similar suggestions were offered in 2012–13, however, additionally, one consultant neonatologist and one consultant obstetrician discussed possible benefits of decision support systems for prompting or reminding staff:

*“…with the new EPAS [Enterprise Patient Administration System]… the very sophisticated electronic, medical notes systems… some of them have automatic prompts… if you’ve admitted someone and said, 28 weeks – da da da, ‘bung!’ – up would come, you know, ‘have you considered mag sulphate for this patient?’… So those sorts of prompts that sit behind the system… it’s all about bringing it up and closing the circle” (CN1-2012/13).*

### Environmental context and resources

This domain encompasses any circumstance of an individual’s situation or environment that encourages or discourages them to perform a task, including: resources such as staff, materials, space, and competing time and task constraints. Almost all participants across both time periods highlighted barriers relating to the environment or resources. Midwives and obstetricians in 2011 and 2012–13 discussed the difficulty of preparing and prescribing the therapy as a barrier: *“probably the main barrier would be the complexity of the administration… It’s not simple” (CO3-2012/13).*

Issues around *“having a separate line for it”* and having to *“draw it all up”* were described. Barriers relating to administration were often discussed in the context of time pressures surrounding imminent preterm birth. One consultant obstetrician in 2012–13 expressed concern that the time taken to prescribe magnesium sulphate could deter junior staff:

*“The doctor has got to write this very complicated set of orders… So people will think “Oh, we just haven’t got time. It’s too complicated, let’s just leave it, it’s just too hard” you know?” (CO3-2012/13).*

The main contextual barrier, however, recognised by each professional group across both time periods, was the unpredictability of preterm birth, and the speed at which preterm labour can occur:

*“Ah, I think the major barrier I’ve experienced is not really knowing whether they are going to go into labour or not and sometimes missing the opportunity to start it, because preterm labour can happen so quickly” (TO5-2011).*

In recognition of the unpredictability of preterm birth and the time taken to prepare magnesium sulphate for administration, a frequent suggestion by midwives, and also by obstetricians, across both time periods, was *“ready made”* syringes:

*“When we draw it up, you know it takes us at least 15 minutes… whereas if it’s already pre-done, amazingly quick! A pre-packed syringe or bag would be great” (MW3-2012/13).*

A desire for *“more accurate ways of predicting preterm deliveries” (TO3-2011)* was expressed across both time periods. The use of *“more fetal fibronectin tests, which doesn’t routinely happen” (TO2-2011)* was suggested as a potential facilitator by one trainee obstetrician in 2011.

Clear and concise, easily accessible guidelines and/or protocols were highlighted by all professional groups, particularly in 2011, as an enabler to timely and correct administration. The potential benefits of incorporating recommendations on use of antenatal magnesium sulphate into a *“prematurity guideline” (TN1-2011)* or *“preterm labour protocol” (TN2-2012/13)* were discussed by two trainee neonatologists. In 2012–13, the need for *“the most user-friendly guideline”* was highlighted, particularly in terms of monitoring, and the simplification of the protocol was recognised as a facilitator:

*“There were the two administration regimens… It is a lot clearer now… It’s easier when there is just one protocol and everyone sticks to it” (TO2-2012/13).*

Other potential contextual or resource barriers, not as commonly discussed across the two time periods, included *“run[ning] out of magnesium sulphate a couple of times” (MW5-2011)* (one midwife, 2011), language barriers (one neonatologist and one obstetrician, 2011; one midwife, 2012–13), and distance, with women being transferred from the country (one midwife, 2012–13). Having *“a stock of it on the labour ward” (CO4-2011)* was recognised by one obstetrician in 2011 as an enabler to timely administration; with one trainee neonatologist in 2011 noting *“It needs to be easily available and I think it is”. (TN1-2011).*

In 2012–13, both midwives and obstetricians discussed, and highlighted as barriers, limitations surrounding magnesium sulphate administration being restricted to the labour ward:

*“There was really a bed block up in the delivery suite on that day that prevented her from being transferred immediately which is what we would usually do. Instead, we were told “No, you’ve got to just keep her on the antenatal ward”… So, that was somebody who missed out.” (TO1-2012/13).*

A lack of communication with the planning of preterm birth between the antenatal and labour ward was also discussed in 2012–13. To overcome such barriers, staff suggested accrediting women’s assessment (emergency service) and antenatal ward midwives to administer magnesium sulphate, to allow the therapy to be given in those service areas.

### Beliefs about consequences

This theoretical domain includes an individual’s belief about positive or negative consequences of providing an intervention that may affect his or her behaviour. Staff from each professional group, across both time periods, discussed that an important enabler was the shared beliefs of health professionals and women that this therapy could improve health outcomes for preterm born infants. It was often discussed that despite potential side effects for the mother, the ‘benefit-risk ratio’ was in favour of treatment:

*“I find that most women want to do the best by their baby and if it’s talking about their brain, people want to go ahead with it… Any drug that helps to reduce complications of preterm birth is fantastic” (MW4-2011).*

In 2012–13, one midwife suggested that women and their partners’ uncertainty around *“whether it’ll be of benefit or risk”* could serve as a potential barrier, and identified the benefits of informing families of *“any stats that we’ve got regarding outcomes… That reassures them” (MW7-2012/13).*

One obstetrician in 2011 highlighted a belief of *“not enough hard evidence to support the use of antenatal magnesium sulphate in terms of a good quality [randomised controlled trial] for long term outcomes” (CO2-2011)* as a potential barrier, and believed that *“more long term data needs to be collected.”* In 2012–13 however, one obstetrician and one neonatologist expressed that while good quality evidence existed, there was a need to continue to promote it, due to the absence of immediately visible benefit:

*“It doesn’t have an immediate benefit… you know, not having cerebral palsy in two years’ time… you need to convince them about the statistics and the odds” (CN1-2012/13).*

In 2011, beliefs of a number of obstetricians and one neonatologist that magnesium sulphate could be associated with adverse effects or *“toxicity”* and concern regarding interactions with the tocolytic agent, nifedipine, were recognised as potential barriers. In 2012–13, one trainee obstetrician highlighted *“poor tolerance of the treatment by patients” (TO4-2012/13)* as a possible barrier.

In 2012–13, one trainee obstetrician discussed that uncertainly around timing of birth may lead to hesitation in commencing treatment, especially in the context of being held responsible or ‘blamed’ for potentially unnecessary treatment:

*“… there’s sometimes a culture of, um, blame almost if you start magnesium you sort of get asked the next day “Well, why did you start that? She hasn’t had a baby”” (TO1-2012/13).*

### *Social influences*

This domain refers to the extent to which social influences, including peers and other professional groups, facilitate or hinder (or sometimes both) delivering an intervention. In 2011, one trainee obstetrician discussed that discrepancies between consultants’ opinions served as a potential barrier to administration: *“Sometimes they say no, sometimes they say yes… so we are guided by that as well” (TO4-2011).*

Overwhelmingly, however, across both time periods, staff from all professional groups cited supportive peers as a key facilitator. Midwives highlighted senior staff members, as useful *“to clarify things with” (MW2-2011)* and particularly highlighted benefits of working with registrars, with them being *“really up to speed with it” (MW8-2011).* Midwives also highlighted their own ability to influence colleagues:

*“… working as a team… giving good feedback, to the registrar saying like… “You know, do you think it’s, would be, prudent to order it?”” (MW8-2011).*

Trainee obstetricians across both periods cited midwives and consultant obstetricians as influential in guiding their practice:

*“The fact that the midwives are actually involved in this helps a lot, so if I’ve forgotten, there’s always someone prompting… The consultants are also aware about it so they also, as we’re presenting our plans, they also say “Hey, make sure it’s, it’s part of your management plan”” (TO3-2012/13).*

Senior obstetricians across both periods highlighted positive influences particularly of junior obstetric staff and midwives, but also of neonatologists:

*“… the midwives in the labour ward are pretty good on this, they’re on the ball… And the registrars are too… they usually say well “Do you want the magnesium sulphate with this baby?”… the paediatricians are pretty good at it too” (CO2-2012/13).*

One neonatologist in 2011 suggested that in order to increase uptake you need “*influential people pushing it… someone to drive it along… putting some energy into gaining that momentum” (CN1-2011),* and proposed nominating a dedicated obstetrician or midwife within the hospital to serve as a ‘change champion’. Similarly, a consultant neonatologist in 2012–13 recognised the benefits of having *“key people driving it” (CN1-2012/13).*

A further enabler to increased use, recognised by one neonatologist in 2011, were the NHMRC and Royal Australian and New Zealand College of Obstetricians and Gynaecologists (RANZCOG) endorsements of the guidelines: *“pushing out these guidelines… as accepted best practice, in order for them to be extensively used” (CN3-2011).*

### Professional role and identity

This domain includes an individual’s perceptions about his or her professional role or identity, and how these perceptions or beliefs influence the delivery of an intervention. In 2011, two trainees and two consultant neonatologists described magnesium sulphate as an ‘obstetric medication’ with neonatal benefit, which could serve as a barrier *“we probably don’t push it as hard as we should” (CN1-2011).* In 2012–13, there was a similar recognition by some neonatologists that: *“it’s mainly an obstetric decision” (TN2-2012/13).* Neonatologists in 2012–13 suggested that advising their colleagues to administer antenatal magnesium sulphate was outside of their professional role:

*“I do admit that it’s, it’s very likely a neonatologist will deal with just the baby side of things and the timing of delivery, and not perhaps be proactive about giving the magnesium” (CN3-2012/13).*

### Reinforcement

This theoretical domain relates to factors that would increase the probability of a response, such as rewards, incentives or punishments. In 2011, one trainee and one consultant neonatologist suggested that audit and feedback could serve as an enabler:

*“I mean an audit cycle would be useful… see how many were eligible and how many received it… and using that as a basis to sort of re-educate people… perhaps every single patient, that um, comes in, that is eligible for NICU should, be prospectively audited… “Yes? No? Why not?”” (TN1-2011).*

In 2012–13, one neonatologist similarly suggested that an enabler may be the development of a key performance indicator (KPI) for institutions which could include the utilisation of new findings from clinical trials, such as regarding antenatal magnesium sulphate for fetal neuroprotection. The use of KPIs was likewise mentioned as a potential facilitator by one consultant neonatologist in 2011.

Two obstetricians in 2012–13 suggested incentives to enable improved uptake; one trainee obstetrician ‘joked’that appropriate administration of the therapy could be *“a requirement for getting onto the training program” (TO2-2012/13).* One consultant obstetrician recommended further involving and rewarding staff for their participation in research work related to antenatal magnesium sulphate:

*“… you know, those sorts of things would be an added incentive… Actually, include them in on the thing and say, ok, you’re the key players in this, um, we want you to be part of the paper… you’ll find that those people will respond accordingly because it’s like waving a carrot in front of them” (CO1-2012/13).*

## Discussion

Our study has provided valuable data on barriers and enablers to evidence-based implementation of antenatal magnesium sulphate for fetal neuroprotection, with a number of key findings. First, self-reported use and knowledge of this therapy appeared to increase over time. Second, barriers were largely common across professional groups and over time, and most often related to the ‘environmental context and resources,’ with gaps in ‘knowledge’ and ‘skills’ less common, particularly in the later time period. Third, diverse enablers were identified by all professional groups which could be used to change their and their colleagues’ practice.

In regards to self-reported administration or advice for administration of antenatal magnesium sulphate, obstetricians, neonatologists and midwives in 2012–13, compared with in 2011, were more likely to report using/advising use in their clinical practice. While our study was not designed to statistically compare use across time periods, the apparent increase in self-reported use is supported by quantitative findings from audit studies at our institution, and others [25, 26], which have revealed, for example, an increase in uptake from approximately 30 % in 2010 to almost 80 % in 2012–13 [22, 27].

Using the TDF proposed by Michie and colleagues [17, 18], we assessed barriers and enablers to the use of this therapy as perceived by health professionals. The most significant barriers across both time periods related to the domain ‘environmental context and resources’, and included the difficulty to predict preterm birth and the complex administration process, particularly within the context of time pressures surrounding unexpected or rapidly progressing preterm labour. In a recent retrospective audit of ‘non-receipt’ of this therapy at our institution, non-receipt was similarly determined to be associated most often with immediately imminent or indicated emergent birth [27].

Not surprisingly, a common suggestion or enabler to increase uptake was the use of pre-drawn syringes. The use of ready-made solutions has importantly been recognised as one strategy to reduce medication errors associated with magnesium sulphate [28]. In 2012–13, a further facilitator to timely administration recognised by a number of staff members was the expansion of the service areas where magnesium sulphate could be safely administered to include the women’s emergency service and antenatal ward.

Other prominent barriers identified across both time periods related to the domains ‘knowledge’ and ‘skills’ and ‘memory, attention, and decision processes’, including: a lack of awareness of the guideline/protocol, unfamiliarity with the processes for administration, and ‘forgetting’. While lack of awareness was identified more commonly as a barrier in 2011, across both time periods, education was identified as an important enabler. However in 2012–13, an emphasis was placed on the need for continued education for reinforcing knowledge, particularly in the context of frequent staff ‘turn-over’. To overcome the barrier of not remembering, in 2011 participants often suggested a need for visual reminders (posters). In 2012–13, participants suggested using electronic support systems to aid in ‘flagging’ this treatment for potentially eligible women.

A key enabler identified by participants across both time periods (relating to the domain ‘beliefs about consequences’) was staff and families’ belief in the ability for this therapy to improve health outcomes. Arguably the most significant enabler discussed by all professional groups, however, was the support from other staff members (associated with the ‘social influences’ domain). All professional groups – midwives, obstetric trainees and consultants, and neonatal trainees and consultants – were recognised by their colleagues as having influential roles in guiding practice and facilitating the delivery of this intervention.

Our study highlighted barriers and enablers that were both common and differing across two time periods. It is possible that some of the changes over time in perceived barriers/enablers can be partially attributed to the availability of WISH materials following the first phase of interviews – including posters that were displayed in delivery suite, antenatal ward and emergency service areas, health professional information sheets that were widely distributed, and in-service sessions that were regularly held [15]. Certainly, increased awareness of the guidelines was observed in the second time period, when participants focused on the need to reinforce knowledge along with other new approaches to increase uptake. WISH Project strategies have also included the identification of a local ‘clinical champion’ at our institution, whose role has been to offer support to and advise colleagues [15]. Encouragingly, participants in this study discussed the importance of such leaders to drive implementation and act as a ‘catalyst’.

Our study had some limitations. Firstly, study participants were recruited from only one tertiary maternity centre in South Australia. Additional barriers and enablers may exist in other settings and locations, which would be important to identify if developing tailored interventions for addressing evidence-practice gaps [17]. However, many of the barriers and enablers (and corresponding theoretical domains) identified in our study are likely to be common, and thus our findings are useful to health professionals in other maternity centres wishing to improve the use of this neuroprotective treatment for very preterm infants.

Secondly, the number of health professionals interviewed in each group of our study was somewhat arbitrarily pre-specified; and in the second time period, we had fewer participants than expected. However, as no new information was forthcoming, we believe data saturation was achieved. Interviews with women at risk of very preterm birth, reception/administration staff, and staff working at common referral hospitals, would, however also provide valuable data, and these groups could be the subject of additional research.

Finally, we did not, as some previous barrier and enabler exploration studies have done [19, 21], use the TDF to guide the interview schedule, and individual questions were not framed in order to explore each domain of the framework individually. The TDF, used during the coding/analysis stage, was valuable, with benefits of using the framework including the efficiency with which the interview data could be coded. We also believe that the open-ended nature of relevant questions in this study should not have biased barriers/enablers from any particular domain emerging.

## Conclusions

Our study, using the TDF, has provided valuable data on barriers and enablers to evidence-based implementation of antenatal magnesium sulphate for fetal neuroprotection as perceived by obstetricians, neonatologists and midwives. Health professionals identified difficulty in predicting preterm birth, which may be precipitous or immediately imminent, within the context of time constraints and complex administration procedures as key barriers. Regarding enablers to implementation, participants commonly suggested education, reminders, audit and feedback, decision-support systems, expanding the range of services areas, pre-prepared syringes, and support from colleagues or influential individuals. Results of this study will guide the planning and modification of further implementation strategies with the goal of improving neuroprotection for very preterm infants. We encourage research into barriers and enablers in difference settings; some may prove common and others may be setting specific. Seeking pregnant women’s views of barriers and enablers to use of magnesium sulphate for neuroprotection is also important.

## Additional files

Additional file 1:
**Summary of clinical recommendations for antenatal magnesium sulphate prior to preterm birth for neuroprotection of the fetus, infant and child.** (PDF 80 kb) Additional file 2:
**Interview schedule.** (PDF 243 kb)

## Abbreviations

NHMRC: National Health and Medical Research Council
TDF: Theoretrical Domains Framework
WCH: Women's and Children's Hospital
WISH: Working to Improve Survival and Health for babies born very preterm
