# Supplementary material for: Barriers and enablers to implementing antenatal magnesium sulphate for fetal neuroprotection guidelines: a study using the theoretical domains framework
Source: BMC Pregnancy Childbirth. 2015 Aug 18;15:176. doi: 10.1186/s12884-015-0618-9 (PMC4539663; doi:10.1186/s12884-015-0618-9)
Supplement: Additional file 1: — Summary of clinical recommendations for antenatal magnesium sulphate prior to preterm birth for neuroprotection of the fetus, infant and child. (PDF 80 kb) [file 12884_2015_618_MOESM1_ESM.pdf]

Additional File 1, Table 1: Summary of clinical recommendations for antenatal magnesium sulphate prior to preterm birth for neuroprotection of the fetus, infant and child

Clinical Recommendations	Grade^	Guideline Chapter
In women at risk of early preterm* imminent# birth, use magnesium sulphate for neuroprotection of the fetus, infant and child:<br>*when gestational age is less than 30 weeks.<br>#when early preterm birth is planned or definitely expected within 24 hours. (When birth is planned, commence magnesium sulphate as close to four hours before birth as possible).	A	4-7
	B	8
	A	9
• intravenously with a 4 gram loading dose (slowly over 20-30 minutes) and 1 gram per hour maintenance dose via the intravenous route, with no immediate repeat doses. Continue regimen until birth or for 24 hours, whichever comes first.	C	10
• regardless of plurality (number of babies in utero).	B	11
• regardless of the reason the woman (at less than 30 weeks' gestation) is considered to be at risk of preterm birth.	B	12
• regardless of parity (number of previous births for the woman).	B	13
• regardless of anticipated mode of birth.	B	14
• regardless of whether or not antenatal corticosteroids have been given.	B	15

^These grades are based on the Australian National Health and Medical Research Council (NHMRC) standards for grading recommendations for developers of guidelines
