# Supplementary material for: Barriers and enablers to implementing antenatal magnesium sulphate for fetal neuroprotection guidelines: a study using the theoretical domains framework
Source: BMC Pregnancy Childbirth. 2015 Aug 18;15:176. doi: 10.1186/s12884-015-0618-9 (PMC4539663; doi:10.1186/s12884-015-0618-9)
Supplement: Additional file 2: — Interview schedule. (PDF 243 kb) [file 12884_2015_618_MOESM2_ESM.pdf]

Study ID: _______

WISH Project Interview Schedule

Working to Improve Survival and Health for babies born very preterm

Date completed: ____

Title (position in organisation):

- Consultant obstetrician: ❑
- Trainee medical officer (obstetric): ❑
- Consultant neonatologist: ❑
- Trainee medical officer (neonatal): ❑
- Midwife: ❑

How long have you been working at the WCH?_________________________________

Introduction

"This interview is designed to gain an understanding of your knowledge and the current use of magnesium sulphate within your hospital. The interview is being recorded as I do not want to miss any of your comments. All names will be de-identified or removed from the typed transcripts and the actual recording will be destroyed. Your responses are therefore confidential. The interview will take approximately 10 minutes."

1. Do you administer magnesium sulphate to women at risk of preterm birth?

   OR

   Do you advise your colleagues to administer magnesium sulphate to women at risk of preterm birth?

   ❑ Routinely ❑ Sometimes ❑ Infrequently ❑ Never

1.1. If ***Yes*** (***routinely or sometimes***), please describe in which situations and how (considering dosage, timing, gestational age, and reason(s) for preterm birth):

Dosage____

____

____

Timing prior to birth____

____

____

Gestational age____

____

____

Reason(s) for preterm birth____

____

____

1.2 If ***No*** (***infrequently or never***), why?

2. What are your views on clinical practice guidelines in general?

3. How do you keep up-to-date with current clinical practice guidelines?

4. Are you aware of the *'Antenatal magnesium sulphate prior to preterm birth for neuroprotection of the fetus, infant and child: national clinical practice guidelines?'*

❑ Yes ❑ No

4.1 If ***Yes***, can you describe the key clinical recommendations of these guidelines?

[If ***No***, read out the recommendations within the box below]

The guidelines recommend that magnesium sulphate be administered to women at risk of early preterm, imminent birth, when gestational age is less than 30 weeks, when early preterm birth is planned or definitely expected within 24 hours. The magnesium sulphate should be administered intravenously with a 4 gram loading dose (slowly over 20-30 minutes) and a 1 gram per hour maintenance via IV route, with no immediate repeat doses, and continued until birth or for 24 hours whichever comes first.

5. What do you think would be the benefits of using magnesium sulphate as stated in the guidelines?

6. Do you have any concerns about possible adverse effects of magnesium sulphate administration?

7. Do your colleagues prescribe magnesium sulphate to women at risk of preterm birth?

❑ Yes ❑ No ❑ Unsure

7.1 If ***No***, why not?

8. Can you describe any barriers for you and/or your colleagues administering or advising administration of magnesium sulphate to women at risk of preterm birth?

____
____
____

8.1 What would help overcome these barriers?

____
____
____

9. What would you suggest would enable you and/or your colleagues improve the uptake of magnesium sulphate in line with the clinical practice guideline recommendations?

____
____
____

10. Do you have any other comments?

____
____
____
